# Ultrasound transducer disinfection for percutaneous procedures: A review of the evidence supporting guideline recommendations

**DOI:** 10.1002/ajum.12408

**Published:** 2024-09-30

**Authors:** Nathan Peters, Frances Williamson, Victoria Eley

**Affiliations:** ^1^ Department of Anaesthesia and Perioperative Medicine Royal Brisbane and Women's Hospital Brisbane Queensland Australia; ^2^ Faculty of Medicine The University of Queensland Brisbane Queensland Australia; ^3^ Emergency and Trauma Centre Royal Brisbane and Women's Hospital Brisbane Queensland Australia

**Keywords:** disinfection, infection control, ultrasonography

## Abstract

**Introduction/Purpose:**

There are varying international recommendations regarding the minimum level of disinfection required for ultrasound transducers used in percutaneous procedures. While some guidelines recommend high‐level disinfection (HLD), others question the additional benefit this delivers over low‐level disinfection (LLD).

**Methods:**

This narrative review identifies current guidelines and evaluates the evidence used to support disinfection recommendations for ultrasound transducers used in percutaneous procedures. Thirteen guidelines were identified using a search encompassing PubMed, Embase, Scopus and Google from 1st January 2013 to 31st January 2024.

**Results:**

No guidelines were supported by high‐quality evidence, instead, guidelines relied upon: expert opinion through the application of national standards and infection control principles; consideration of recommendations from other published guidelines; and the incidence of infection from retrospective studies. Guidelines were uniformly supportive of using ultrasound transducer covers and sterile ultrasound gel during ultrasound‐guided percutaneous procedures. However, the minimum recommended disinfection level was varied with seven guidelines recommending HLD, four LLD and two not specifying a level. Spaulding's classification was commonly used to support disinfection recommendations, however, the resultant wide variation in classification and subsequent recommendations suggest that its utility in accurately determining the minimum level of disinfection in this specific context is low.

**Conclusion:**

Without high‐level evidence, using a risk‐based assessment will likely remain fundamental to future guideline recommendations in determining the minimum disinfection level for an ultrasound transducer used in percutaneous procedures. This risk assessment must include the highest level of evidence available in addition to acknowledging the contribution of all steps taken to prevent infection during ultrasound‐guided percutaneous procedures.

## Introduction

Ultrasound‐guided percutaneous procedures involve introducing a needle through skin towards a target region contained within the field of view of an ultrasound transducer.^1^ In most percutaneous procedures, the ultrasound transducer is applied externally to intact skin with the needle puncture site occurring within close proximity (e.g. vascular access, therapeutic and diagnostic injections/aspirations and biopsies). Ultrasound guidance is the accepted standard of care for many of these procedures due to improvements in procedural success as well as a recognised reduction in complications.^2^, ^3^, ^4^

As a reusable medical device, the ultrasound transducer must be reprocessed, which involves cleaning and disinfection, prior to the next use.^5^ This process aims to minimise the risk of infection to the subsequent patient from the device. Spaulding's classification was first proposed in the 1950s and remains central to many national standards and infection control guidelines.^6^ This three‐tier system uses the level of infectious risk based on the type of human tissue that the reusable medical device is to be next used upon in order to determine the level of disinfection required (Table 1).^5^, ^6^, ^7^

**Table 1:** Spaulding's classification system for reusable medical devices.^5^

Level of risk	Definition	Minimum level of disinfection
Critical	Intended to be introduced directly into or have contact with the vascular system or normally sterile areas of the body	Sterilisation
Semi‐critical	Medical device that comes into contact with mucous membranes or non‐intact skin	High‐level
Non‐critical	Medical device that comes into contact with intact skin but not mucous membranes	Low‐level

**Figure 1:**
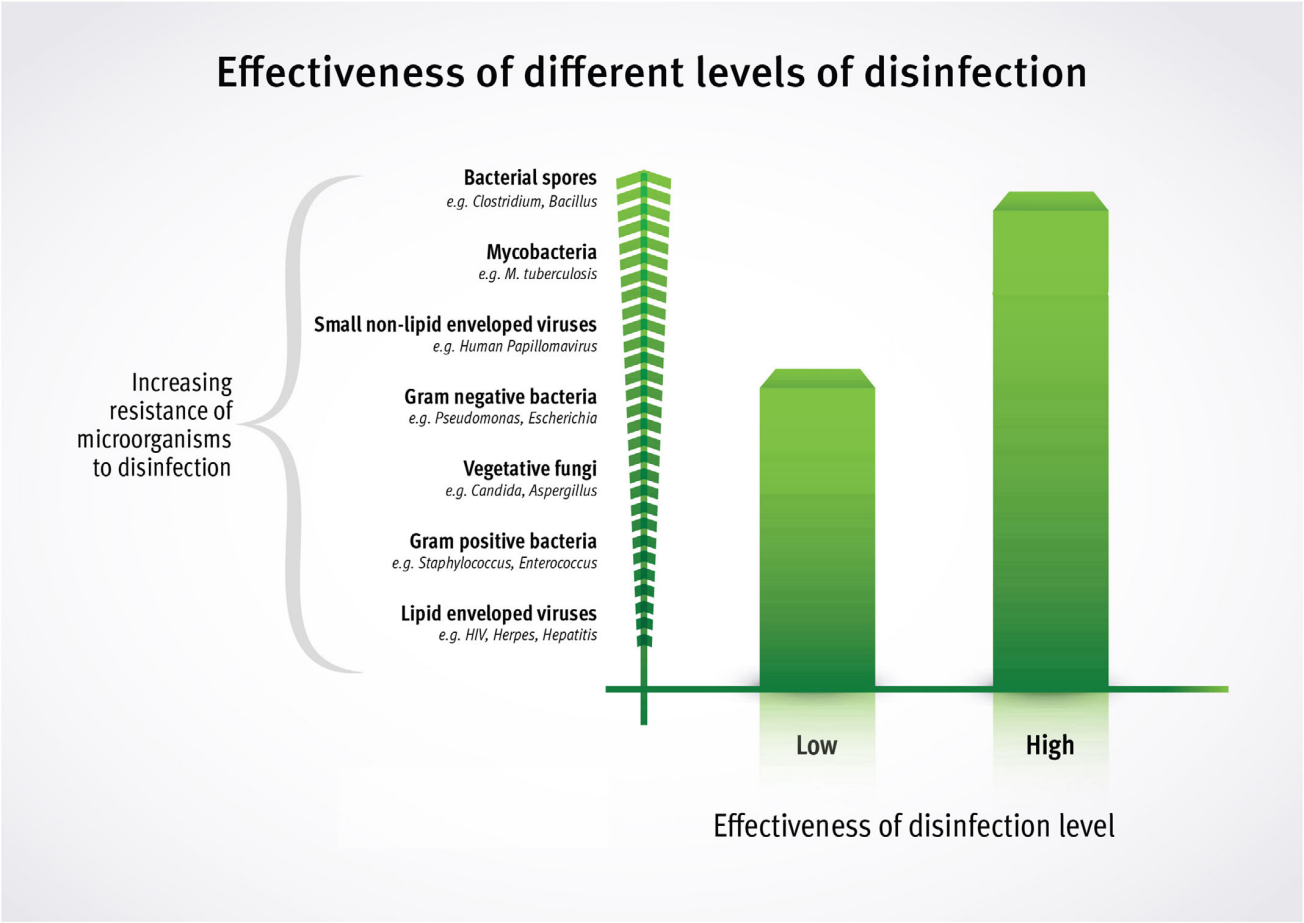
Effectiveness of different levels of disinfectants across a spectrum of different microorganisms.^7^, ^12^

National and international debate continues over what level of disinfection is required for ultrasound transducers used as part of a percutaneous procedure, with some guidelines recommending high‐level disinfection (HLD) and others low‐level disinfection (LLD).^1^, ^8^, ^9^, ^10^, ^11^ In comparison to LLD, HLD is effective against microorganisms that are more resistant to disinfection such as mycobacteria, non‐lipid enveloped viruses and bacterial endospores (Figure 1).^7^, ^12^ In contrast, the microorganisms known to cause infection related to percutaneous procedures, such as vascular access, are vegetative gram‐positive and gram‐negative bacteria and fungi (*Candida* spp.) which are within the spectrum of activity for both low‐ and high‐level disinfectants.^13^, ^14^, ^15^, ^16^, ^17^, ^18^ Given the high frequency ultrasound guided percutaneous procedures are performed in current medical pracitce, concerns have been raised about expectations of HLD negatively impacting on patient care by limiting the availability of ultrasound equipment owing to the higher cost required to establish and maintain HLD processes compared to LLD.^1^, ^9^, ^11^, ^19^ In previsous research from our group, using Peters *et al*.^19^ as a comparative example of cost, the HLD system used in that study was in the order of 130 times more expensive than LLD per patient ($13.20 AUD vs. $0.10 Australian dollars (AUD). Given the impact these disinfection recommendations have on patients, staff and healthcare organisations, this review aims to explore the quality of evidence used by guidelines to support disinfection recommendations for ultrasound transducers used in percutaneous procedures.

## Methods

A literature search was conducted to identify ultrasound transducer disinfection guidelines which included recommendations relating to percutaneous procedures between 1 January 2013 and 31 January 2024. PubMed, Embase and Scopus were used to perform the search. Search terms were limited to title and abstract and included: ‘Ultrasonography OR Transducers’; ‘Cross Infection/prevention and control’; ‘Equipment Contamination’; ‘Medical device contamination’; ‘Infection control’; and ‘Cleaning OR Reprocessing OR Disinfection’. An advanced Google search was also undertaken using the ‘find pages with all these words’: function with ‘ultrasound disinfection guideline’, which was limited to .PDF file type. The inclusion criteria were any national or societal guideline written in English that contained recommendations for ultrasound‐guided percutaneous procedures. Industry‐sponsored advertisements and documents were excluded from the results. All studies identified by the search were collated with titles and abstracts reviewed by the first author to identify relevant publications. Recommendations made were noted as well as the methods used to support these, including any level of evidence grading systems.

## Results

Thirteen published guidelines on ultrasound transducer disinfection were identified for inclusion. Methods used to support guideline recommendations included: Spaulding's classification; previously published guidelines; national infection control standards; research publications relating to percutaneous procedure infection rates or other related markers of infectious risk; and evidence grading or agreement systems. In 10 guidelines analysed, additional recommendations aimed at minimising infection risk during percutaneous procedures were noted which included the use of ultrasound transducer covers and sterile ultrasound gel (Table 2).

**Table 2:** A summary of international guideline recommendations for ultrasound‐guided percutaneous procedures.

Year	Country or area	Organisation	Ultrasound transducers used in percutaneous procedures				
			Disinfection level and supportive evidence			Ultrasound gel	Transducer cover use
			Minimum level of disinfection	Spaulding's classification	Research relating to infection		
2024	Canada	Canadian Association of Emergency Physicians^20^	Low‐level	Not categorised	Yes	Sterile	Yes – Sterility dependent on procedure
2023	United States of America	American Institute of Ultrasound in Medicine^21^	Low‐level	Not categorised	Yes	Sterile	Yes – Sterility dependent on procedure
2023	Canada	College of Physicians and Surgeons of British Columbia^22^	High‐level	Critical	No	Sterile	Yes – Sterile
2022	Canada	Infection prevention and control Canada^23^	High‐level	Critical	No	Sterile	Yes – Sterile
2020	United States of America	Society of Diagnostic Medical Sonography^24^	Low‐level^b^	Non‐critical	No	Sterile	Yes – Sterility dependent on procedure
2020	World	World Federation for Ultrasound in Medicine and Biology^25^	High‐level	Critical	No	Sterile	Yes – Sterile
2020	Australia	College of Intensive Care Medicine^26^	High‐level	Critical	No	Sterile	Yes – Sterile
2020	England	British Medical Ultrasound Society^27^	Not specified^a^	Semi‐critical	No	Not specified	Not specified
2018	United States of America	American College of Emergency Physicians^28^	Low‐level	Non‐critical	Yes	Sterile	Yes – Sterile
2018	Germany	German Society of Ultrasound in Medicine^29^	Not specified^a^	Not categorised	Yes	Sterile	Yes – Sterile if contacting puncture site
2017	Scotland	National Health Service^30^	High‐level	Semi‐critical	No	Not specified	Not specified
2017	Europe	European Society of Radiology^31^	High‐level	Critical	No	Sterile	Yes – Sterile
2017	Australia	Australasian Society for Ultrasound in Medicine^32^	High‐level	Semi‐critical	Yes	Sterile	Yes – Sterile

^a^ Lack of regulatory recognition of low‐ or high‐level disinfectants in this country.

^b^ High‐level preferred.

Ten of the thirteen guidelines categorised ultrasound transducers used in percutaneous procedures according to Spaulding's classification system. Of the three levels of risk Spaulding considered, five guidelines considered these transducers critical, three guidelines semi‐critical and two guidelines non‐critical. Of the eight guidelines that considered these ultrasound transducers semi‐critical or critical, seven recommended HLD with the other not specifying a level of disinfection. LLD was recommended in four of the five guidelines where transducers were classified as non‐critical or not categorised according to Spaulding's with one guideline not specifying a level of disinfection. The two guidelines that did not specify a level of disinfection did so due to local regulations not recognising LLD and HLD when describing disinfectant systems.

Eleven guidelines referenced previously published guidelines, with the exceptions being the National Health Service of Scotland and the Australasian Society for Ultrasound in Medicine. Ten guidelines referred to national standards when developing their recommendations with three organisations not referencing national standards (The Canadian Association of Emergency Physicians, the World Federation for Ultrasound in Medicine and the European Society of Radiology). Examples of the national standards used included the Australian and New Zealand Standards, the German Protection Against Infection Act, the Canadian Standards Association, the Quality Standards for Imaging Standards in the United Kingdom, the Centre for Disease Control in the United States of America and the National Health Service in Scotland. These national standards generally focused on infection prevention and control principles and required interpretation and application to ultrasound transducers by the working groups.

Five guidelines incorporated either published evidence of infection, or other related markers, for percutaneous procedures into their recommendations. The Canadian Association of Emergency Physicians (CAEP) referenced work from Peters *et al*.,^19^ and both they, and the Australasian Society for Ultrasound in Medicine, referenced Cervini *et al*.^33^ In Peters *et al*.^19^ we reported a randomised controlled trial demonstrating that LLD was non‐inferior to HLD in the elimination of pathogenic microorganisms from externally applied ultrasound transducers. Cervini *et al*.^33^ performed a retrospective review of over 13,500 ultrasound‐guided interventions (e.g. fine‐needle aspiration, biopsy and drain placement) and identified the incidence of infection as 0.1% (14/13,534). The American Institute of Ultrasound in Medicine (AIUM) and the American College of Emergency Physicians (ACEP) both used work from Adhikari *et al*.^34^ which retrospectively compared infection rates between ultrasound‐guided and traditionally placed peripheral intravenous cannulas which showed low infection rates for both arms (0.52% ultrasound and 0.78% traditional; P = 0.68).^21^, ^28^ The German Society of Ultrasound in Medicine performed the most extensive review of literature relating to infection in percutaneous procedures including procedures such as injections, biopsies, drain placements, catheter insertions and local tissue ablations. Based on their extensive evaluation of registry data, they found infections after ultrasound‐guided percutaneous interventions in 0.1–0.2% of cases, with complex therapeutic interventions having a slightly higher rate.^29^

The only guideline to incorporate an evidence‐based grading system was the National Health Service Scotland while the German Society for Ultrasound in Medicine used a method of assessing working group agreement to support their recommendations. The National Health Service in Scotland used the Scottish Intercollegiate Guideline Network (SIGN) system to grade the level of evidence used to support some guideline recommendations.^30^ However, they did not apply this to ultrasound transducers used in percutaneous procedures, instead relying on Spaulding's classification.^30^ The German Society of Ultrasound in Medicine used a Delphi technique to determine their guideline recommendations. Each recommendation was assessed by all authors and those that did not achieve agreement of at least 75% (12/16 authors) were discarded or revised and then reconsidered.^29^ The recommendations accepted for publication were labelled as ‘consensus’ (agreement of 12–13/16 authors), with ‘strong consensus’ (14–15/16 authors) or unanimous (16/16 authors).

## Discussion

### Level of disinfection for ultrasound transducers

The variability across guideline recommendations reflects an absence of published high‐level evidence. Guidelines from the Canadian Association of Emergency Physicians and the German Society of Ultrasound in Medicine specifically noted the challenges arising from the lack of high‐quality data in developing recommendations.^20^, ^29^ With little available high‐quality data, many recommendations within the guidelines relied on lower‐quality evidence including expert opinion through the application of national standards and infection control principles, consideration of recommendations from other published guidelines, and the incidence of infection from retrospective studies.

Spaulding's classification was identified as a commonly utilised infection control principle‐based decision‐making tool by guidelines. This classification tool was frequently chosen due to its inclusion in other areas of infection control and its presence in national standards. However, the application of Spaulding's system to ultrasound transducers used in percutaneous procedures resulted in differing classifications and disinfection recommendations across the guideline development groups. This poor reproducibility suggests that its utility in accurately determining the level of disinfection required in this specific clinical context is low.

Spaulding's classification system was modified by some guidelines and this may have contributed to the variability across recommendations. Five of the 10 guidelines that used Spaulding's system modified the definition of a semi‐critical item to include the potential risk of contamination with blood or body fluids.^25^, ^26^, ^30^, ^31^, ^32^ However, according to the system Spaulding designed, the only factor that determines how a medical device is reprocessed is its site of next intended use.^5^, ^6^ Contamination present on an ultrasound transducer following use does not influence the level of disinfection as the first step in reprocessing any medical device is to clean it of any organic and non‐organic material.^5^, ^8^, ^35^ Cleaning must be performed irrespective of the level of disinfection to follow, as this manually removes visible contaminants present after use thereby allowing the disinfectant to have direct contact with the device to effectively eliminate the remaining residual microorganisms.^35^ Importantly for ultrasound transducers used in percutaneous procedures, the combination of proper cleaning followed by disinfection, using either low or high level, would effectively eliminate bloodborne viruses (e.g. Hepatitis and HIV) as these large lipid‐enveloped viruses are the most sensitive microorganisms to all levels of disinfection.^7^, ^12^

Many newer guidelines have considered recommendations made in earlier published guidelines. However, it should be recognised that earlier guidelines used little evidence arising from ultrasound‐guided percutaneous procedures to support their recommendations. For example, in 2017, Nyhsen *et al*.^31^ referenced evidence relating to transvaginal ultrasound as well as a letter to the editor which confirmed, in a non‐ultrasound setting, hepatitis C contamination of acupuncture needles was evident after use on hepatitis C‐positive patients. In the same year, Basseal *et al*.^32^ cited a study which demonstrated residual transducer contamination was present when the adherence to cleaning and disinfection protocols was not known. The current relevance of this research when comparing the effectiveness of different levels of disinfection of ultrasound transducers in reducing the infection rate associated with ultrasound‐guided percutaneous procedures is extremely low.

Only 5 of the 13 guidelines considered evidence relating to infection arising from ultrasound‐guided percutaneous procedures in determining the minimum level of disinfection required. One guideline recommended HLD, three recommended LLD and the other lacked a recommendation due to differences in the national regulatory categorisation of disinfectants. Evidence relating to infection was primarily from retrospective data where transducers did not undergo HLD, including a wide variety of clinical percutaneous procedures, and with varying aseptic techniques with and without sterile ultrasound gel and transducer covers. Despite these limitations, it was shown that the infection rates related to ultrasound‐guided percutaneous procedures were very low (0.1–0.5%). In the cases where infection was identified, the causal factors were not further described, and it was unclear if the ultrasound transducer was a contributor or if other patient or procedural factors predominated. Despite this, the Australasian Society for Ultrasound in Medicine recommended HLD in percutaneous procedures while the other remaining four guidelines used these data to question the additional benefit HLD could have over LLD in reducing this already very low incidence of infection.^20^, ^21^, ^28^, ^29^, ^32^

### Ultrasound transducer covers and gel

Recommendations regarding the use of ultrasound transducer covers and sterile gel were frequently incorporated into guidelines. Despite the widespread recommendation to use transducer covers, six guidelines directly question their benefit in preventing infection citing the potential for loss of cover integrity during use.^24^, ^26^, ^27^, ^30^, ^31^, ^32^ Where these statements were referenced, the incidence of transducer integrity loss had commonly been extrapolated from other ultrasound procedures (e.g. transvaginal ultrasound).^31^, ^32^ The appropriateness of this extrapolation for percutaneous procedures remains questionable especially given the small amount of contact that a covered ultrasound transducer has on the patient's skin when compared to more invasive ultrasound examinations. Where addressed in guidelines all recommended ultrasound transducer cover use to minimise infection risk despite the uncertain incidence and significance of loss of transducer cover integrity.

In comparison to medical devices which undergo sterilisation, ultrasound transducers which undergo high‐ or low‐level disinfection are not guaranteed to be free from all viable microorganisms and should therefore not be used on normally sterile areas of the body.^5^ Additionally, their non‐sterile storage and handling following disinfection means that microbial contamination could occur before it is next used.^5^ As a result of this, when performing an ultrasound‐guided percutaneous procedure, additional steps are required to minimise the risk of infection, commonly referred to as aseptic technique. Aseptic technique aims to ensure the absence of pathogenic organisms in sufficient quantity around the puncture site or inserted device to minimise the risk of infection.^36^ If, during an ultrasound‐guided percutaneous procedure the needle insertion site is within the proximity of and/or it is possible that the needle may contact the transducer, a sterile transducer cover is required to maintain aseptic technique.^36^ While three guidelines gave some discretion to the clinician to determine if the transducer cover is required to be sterile, overall, it appears that guidelines are strongly supportive of sterile transducer cover use during ultrasound‐guided percutaneous procedures.

Sterile ultrasound gel was frequently recommended in ultrasound transducer disinfection guidelines. It is foreseeable that during a percutaneous procedure, the device being inserted, and the insertion site, could be contaminated with the surrounding ultrasound gel owing to its proximity. There have also been published cases of infection arising from contaminated gel from bacteria such as *Burkholderia* spp. and *Acinetobacter* spp. Notably, not all outbreaks have been from contamination of multi‐use gel as some arose from contamination at the point of gel manufacture.^37^, ^38^, ^39^, ^40^, ^41^, ^42^, ^43^ As a result, guidelines have uniformly recommended the use of sterile ultrasound gel when performing ultrasound‐guided procedures to minimise the risk of infection.

### Considerations for future ultrasound disinfection guidelines

Disagreement between guideline recommendations has commonly arisen due to differences in the interpretation and application of infection control principles. Applying Spaulding's three‐tier classification system to ultrasound transducers used in percutaneous procedures has resulted in a variety of outcomes and recommendations across multiple guidelines. This highlights its limitation when used as the only method for determining disinfection requirements. Furthermore, more recent international guidelines have attributed less significance to Spaulding's classification in determining the level of disinfection required. Importantly, this change reflects a transition to using a more broadly encompassing infectious risk assessment, which is not only limited to ultrasound transducer disinfection but also considers other additional important infection risk mitigating steps such as the use of sterile gel, transducer covers and aseptic technique when performing an ultrasound‐guided percutaneous procedure.

**Figure 2:**
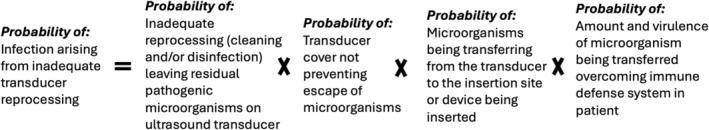
An example of a probability risk assessment for infection arising from an ultrasound‐guided percutaneous procedure due to inadequate ultrasound transducer reprocessing.

Using a risk‐based assessment will remain fundamental in developing recommendations for future guidelines without high‐level evidence becoming available. While there are different risk assessment methods available, a probabilistic risk assessment gives consideration to all risk‐mitigating steps and could be effectively used to determine the infectious risk arising from an ultrasound‐guided percutaneous procedure. This method requires the consideration of how the probability of a fault occurring at each procedural step may contribute to the overall risk of infection.^44^ Examples of these steps include transducer cleaning and disinfection, transducer cover use and integrity, sterility of ultrasound gel used and aseptic technique. This risk assessment method can also be used to better understand the probability of infection arising from an inadequately disinfected transducer used in an ultrasound‐guided percutaneous procedure (Figure 2). In this scenario a sequence of faults is required to occur and it is the product of the probabilities of all these faults, not their individual probability, that determines the infectious risk.^44^ This system provides significant additional protection because if the probability of any one of these faults occurring approaches zero then the impact of inadequate transducer disinfection in contributing to the infection risk also approaches zero.

Fortunately for patients, the risk of infection associated with ultrasound‐guided percutaneous procedures appears very low.^29^ While this speaks to the acceptability of previous practice without HLD it also poses a particular challenge to researchers in comparing different levels of transducer disinfection on infection rates. The resources required to conduct a comparative study using infection rate as the primary outcome, which controlled for the wide variety of patient and procedural factors, would be large and not likely feasible. As such using surrogate markers of infection risk, such as the presence or absence of microorganisms following HLD or LLD, may continue to provide the best available comparative evidence until evidence on infection rates becomes available.^19^ As it is likely that guidelines will continue to use evidence from a variety of different sources, future guidelines should be encouraged to utilise systems to indicate the strength of evidence, or agreement, to support recommendations.

## Conclusion

This review has demonstrated that current recommendations for ultrasound transducer disinfection are limited by a lack of high‐level evidence. This has led to a variety of recommendations based on the interpretation of infection control principles, national standards and procedural infection rates. While the recommendations may differ across guidelines, ultimately the aim remains the same, minimising the risk of infection while maintaining the benefits that ultrasound delivers to patients undergoing ultrasound‐guided percutaneous procedures. Given uncertainty will persist over this issue, it is important that future guidelines base recommendations on the highest level of evidence available and this is communicated using an evidence grading system. This should also include a risk‐based assessment of infection which combines all preventative steps taken during an ultrasound‐guided percutaneous procedure when determining the minimum disinfection level for an ultrasound transducer.

## Authorship statement

I confirm that the authorship listing conforms with the journals authorship policy and that all authors have reviewed and agree with the submission of the current manuscript.

## Funding

No funding was received for the conduct of this project.

## Conflict of interest

All authors declare that they have no relevant conflict of interest in relation to this review.

## Author Contributions

**Nathan Peters:** Conceptualization; writing – original draft; writing – review and editing; methodology; formal analysis; data curation. **Frances Williamson:** Writing – review and editing; methodology. **Victoria Eley:** Writing – review and editing; supervision.
